# Xanthohumol Is a Potent Pan-Inhibitor of Coronaviruses Targeting Main Protease

**DOI:** 10.3390/ijms222212134

**Published:** 2021-11-09

**Authors:** Yuxi Lin, Ruochen Zang, Yanlong Ma, Zhuoya Wang, Li Li, Siyuan Ding, Rong Zhang, Zhiqiang Wei, Jinbo Yang, Xin Wang

**Affiliations:** 1 Institute of Cancer Biology and Drug Screening, School of Life Sciences, Lanzhou University, Lanzhou 730000, China; linyx18@lzu.edu.cn; 2 Center for Innovation Marine Drug Screening and Evaluation, Pilot National Laboratory for Marine Science and Technology, Qingdao 266237, China; lili5890@ouc.edu.cn; 3 Department of Molecular Microbiology, School of Medicine, Washington University, St. Louis, MO 63110, USA; ruochenzang@wustl.edu (R.Z.); siyuan.ding@wustl.edu (S.D.); 4 Key Laboratory of Marine Drugs of Ministry of Education, School of Medicine and Pharmacy, Ocean University of China, Qingdao 266003, China; 5 Key Laboratory of Medical Molecular Virology (MOE/NHC/CAMS), Shanghai Institute of Infectious Disease and Biosecurity, School of Basic Medical Sciences, Shanghai Medical College, Fudan University, Shanghai 200032, China; 18111010054@fudan.edu.cn (Y.M.); rong_zhang@fudan.edu.cn (R.Z.); 6 Center for High Performance Computing and System Simulation, Pilot National Laboratory for Marine Science and Technology, Qingdao 266237, China; zywang3@qnlm.ac (Z.W.); zqwei@qnlm.ac (Z.W.); 7 Marine Biomedical Research Institute of Qingdao, Qingdao 266071, China

**Keywords:** Xanthohumol, natural product, SARS-CoV-2, PEDV, coronavirus

## Abstract

Coronaviruses cause diseases in humans and livestock. The SARS-CoV-2 is infecting millions of human beings, with high morbidity and mortality worldwide. The main protease (M^pro^) of coronavirus plays a pivotal role in viral replication and transcription, which, in theory, is an attractive drug target for antiviral drug development. It has been extensively discussed whether Xanthohumol is able to help COVID-19 patients. Here, we report that Xanthohumol, a small molecule in clinical trials from hops (*Humulus lupulus*), was a potent pan-inhibitor for various coronaviruses by targeting M^pro^, for example, betacoronavirus SARS-CoV-2 (IC_50_ value of 1.53 μM), and alphacoronavirus PEDV (IC_50_ value of 7.51 μM). Xanthohumol inhibited M^pro^ activities in the enzymatical assays, while pretreatment with Xanthohumol restricted the SARS-CoV-2 and PEDV replication in Vero-E6 cells. Therefore, Xanthohumol is a potent pan-inhibitor of coronaviruses and an excellent lead compound for further drug development.

## 1. Introduction

Coronaviruses (CoVs) infect mammals and birds, leading to severe infectious diseases in rare cases. Most human coronavirus (hCoV) infections cause no, or mild, symptoms, but some become fatal, such as SARS-CoV-2, SARS-CoV, and MERS-CoV [1,2]. Animal coronaviruses lead to gross economic losses, with examples including the porcine epidemic diarrhea virus (PEDV), and the transmissible gastroenteritis virus (TGEV) [3]. To September 2021, SARS-CoV-2 has caused over 200 million infections and 4.58 million deaths worldwide. Vaccines have largely controlled viral infections, but various emerging variants are escaping the neutralization by vaccines. On the other hand, some coronaviruses infecting livestock have been reported to be zoonotic [4,5], which may be the origin for the next breakout. It is highly desirable to screen pan-inhibitors for emerging coronaviruses.

Coronaviruses are large positive-strand RNA viruses, with a unique life cycle, belonging to the Coronaviridae family [6]. After entry, viruses immediately translate two polyproteins, pp1a (~450 kDa) and pp1ab (~750 kDa), using their genomic RNA, and generate 12–16 individual nonstructural proteins (NSPs) by the cleavages of the main protease (M^pro^ or 3CLpro) and papain-like protease (PLpro). NSPs assemble into viral replication machinery, taking part in various critical processes, such as genome duplication, protein translation, and immune evasion [6]. To be noted, these viral proteins are highly conserved in the Coronaviridae family, which means chemicals targeting these proteins might inhibit multiple coronaviruses. Thus, people initially expected that the RdRp (RNA-dependent RNA polymerase) inhibitor, Remdesivir (GS-5734), could cure COVID-19 patients because its parent chemical, GS-441524, displayed antiviral activities against other CoVs [7,8]. Unfortunately, the unsuccessful clinical trials of Remdesivir for COVID-19 remind us that screening novel antiviral agents remains attractive, especially for pan-inhibitors against coronaviruses [9].

M^pro^ is a viral-encoding cysteine protease [10]. Protease inhibitors are attractive candidates for antiviral drug development. Many protease inhibitors have been marketed as antiviral agents (i.e., Boceprevir for HCV, Darunavir for HIV). Because coronaviral M^pro^ is distinct from all human proteases [10], and critical in the viral life cycle, compounds that suppress M^pro^ activities may be pan-inhibitors of coronaviruses without side effects. The first generation of M^pro^ is the covalent inhibitor, N3, which mimics the native substrate peptides with additional Michael acceptors as warheads [11]. More and more peptidic inhibitors/peptidomimetics have been developed with amazing activities and low toxicities, for example, GC-376, 11a, and 11b [12,13]. Moreover, M^pro^ inhibitors have also been found via high-throughput screening approaches. Ebselen, PX-12, and carmofur may covalently interact with the C145 residue of M^pro^, which is critical to its catalytical activities [14]. Pfizer has announced the launch of a phase I clinical trial with PF-07321332 [15]. Until now, it is the first M^pro^ inhibitor in a clinical trial, and we are expecting the coming clinical results.

Plants are excellent resources for protease inhibitor discovery. Shikonin, a natural product from herbs, is a potential M^pro^ inhibitor against SARS-CoV-2 [14]. Phenolic compounds (PCs) from plants and their derivatives present antiviral activities against HSV-1, HIV, HCV, and others [16]. Phenolic compounds (PCs) are generally found in fruits, vegetables, herbs, flowers, and seeds, including phenolic acids and flavonoids (flavonols, catechins, flavones, chalcones, etc.). Xanthohumol is a prenylated chalcone from the hop plant (*Humulus lupulus*) that contributes to the bitterness in beer. It has been highly analyzed by researchers as an antiviral agent or antioxidant [17]. It has been reported to inhibit cervical cancer, colorectal cancer, and prostate cancer [18,19,20,21]. Our previous works found that Xanthohumol regulated the Th1/Th2 balance in a tumor model [22]. Xanthohumol inhibits HSV-1 with an IC_50_ value of 2.7 μg/mL, HIV with an IC_50_ value of ≈20.74 μg/mL, and CMV with an IC_50_ value of 2.5 μg/mL [23,24]. Because of its potent utilization of its antioxidant and anti-inflammatory properties, Xanthohumol has been approved in clinical studies for the safety and subjective tolerability in healthy adults (NCT03735420) [25]. After the breakout of COVID-19, many researchers keep discussing the potent roles of Xanthohumol against SARS-CoV-2. In this study, we found that Xanthohumol inhibited M^pro^, and that it was a potent pan-inhibitor against various coronaviruses. Xanthohumol restricted SARS-CoV-2 and PEDV in vitro. This suggests the potential of Xanthohumol as a coronavirus M^pro^ inhibitor.

## 2. Results

### 2.1. Coronaviral M^pro^ Is Highly Conserved

**Figure 1:**
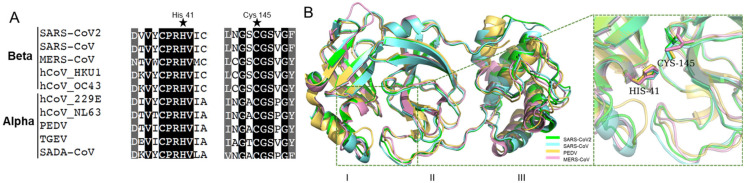
The conserved amino acid sequences and 3D structures of the M^pro^ catalytic domains for different coronaviruses. (**A**) Alignment of neighbor residues on both flanks of the pivotal residue His41, Cys145. (**B**) The three-dimensional (3D) structures of M^pro^ are highly conserved.

M^pro^ contains three conserved domains: I, II, and III. Domain I and II are closely interacted to form a cleft, forming a catalytic core and substrate binding sites. His-41 in Domain I, and Cys-145 in Domain II, are catalytic dyads [10,26]. As shown in Figure 1A, His-41, Cys-145, and their neighbor residues were highly conserved in alpha-coronaviruses (i.e., PEDV and TGEV) and beta-coronaviruses (i.e., SARS-CoV and SARS-CoV-2). Moreover, the three-dimensional (3D) structures were similar for various M^pro^ (Figure 1B), and the amino acid sequences of Domain I and II were consistent in pathogenic coronaviruses (i.e., SARS-CoV-2, SARS-CoV, PEDV, and MERS-CoV) (Figure S1). This suggests that M^pro^ is a promising drug target for pan-inhibitor screening against coronaviruses. Interestingly, the cysteine protease inhibitor, GC376, is a potent M^pro^ inhibitor that shows antiviral activities against SARS-CoV-2, SARS-CoV, and feline coronavirus (FCoV) [27,28]. A recent study developed a pan-inhibitor of M^pro^ restricting multiple coronaviruses in vitro [29]. It suggests that M^pro^ inhibitors might be pan-inhibitors against coronaviruses.

### 2.2. SARS-CoV-2 M^pro^ Inhibitor Screening

To screen M^pro^ inhibitors, recombinant GST-tagged M^pro^ was generated from the E. coli BL21/DE3 strain (CodonPlus, Agilent Technologies Inc., Santa Clara, CA, USA;) using pGEX4 vectors. Since any additional amino acid residues in the N-terminus of M^pro^ could reduce its activity [11,27], tags were removed by Factor Xa (P8010L, New England Biolab, Ipswich, MA, USA) to generate native M^pro^ proteins (Supplementary Figure S2A). The Michaelis constant (Km) was measured to verify the hydrolase reaction. The Km value was 1.10 ± 0.22 μM (Supplementary Figure S2C), referring to a high affinity between the substrates and the enzyme.

**Figure 2:**
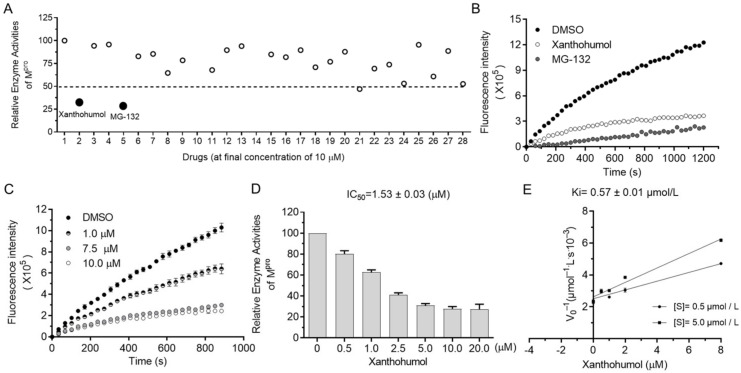
Xanthohumol inhibited hydrolase activities of SARS-CoV-2 M^pro^ in a dose-dependent manner. (**A**) Relative enzyme activities of SARS-CoV-2 M^pro^ in the presence of 10 μM compounds. (**B**) The kinetic curves in the presence of Xanthohumol, MG132, and the solvent, DMSO. Xanthohumol and MG132 were added to the final concentration of 20 μM. (**C**) Xanthohumol inhibited M^pro^ dose-dependently. (**D**) The IC_50_ value of Xanthohumol. (**E**) The Ki value of Xanthohumol on SARS-CoV-2 M^pro^. Data are shown as means ± standard error of mean (SEM) from three independent experiments.

The orchestrated protease system of cells is essential to many biological processes (i.e., misfolded protein degradation, inflammation, antimicrobe invasion, and digestion) that require the low toxicity of protease inhibitors as drug candidates. Since protease inhibitors are abundant in animals and plants, we decided to screen M^pro^ inhibitors from natural products, especially focusing on natural products from foods, food additives, and herbs, which were safe and easily accessible in theory. As shown in Supplementary Figure S2D, more than a hundred natural products were randomly picked from the compound bank and briefly screened at the final concentration of 50 μM. Hits were further tested in the presence of 10 μM compounds, and candidates with an inhibition rate of more than 50% were considered as active. Surprisingly, Xanthohumol and MG132 almost abolished the enzyme activities of M^pro^ at the final concentration of 10 μM (Figure 2A,B). MG132 is a well-established broad-spectrum proteasome inhibitor, used as a spy compound here.

It was found that Xanthohumol reduced M^pro^ hydrolase activities at low concentrations in vitro in a dose-dependent manner (Figure 2C,D). The half inhibition concentration of Xanthohumol on SARS-CoV-2 M^pro^ was 1.53 ± 0.03 μM (Figure 2D). As discussed in our early publications, the IC_50_ value of enzyme inhibitors is closely related to the enzymatical assay. To further analyze the inhibition efficiency of Xanthohumol on SARS-CoV-2 M^pro^, the Ki (the inhibition constant for the inhibitor) value was also measured, which was 0.57 ± 0.01 μM (Figure 2E). It indicated that Xanthohumol reduced SARS-CoV-2 M^pro^ enzymatic activities efficiently in vitro.

### 2.3. Xanthohumol Potentially Inhibits Various Coronaviral M^pro^

**Figure 3:**
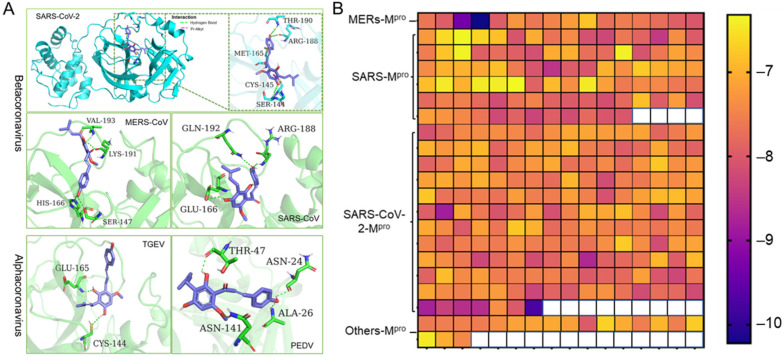
Xanthohumol is a potent pan-inhibitor of coronaviral M^pro^. (**A**) The interaction pattern between Xanthohumol and indicated M^pro^. The M^pro^ was shown in cyan and green. The Xanthohumol was presented in purple. (**B**) The affinity diagrams between Xanthohumol and different M^pro^. M^pro^ structures were downloaded from PDB, and the PDB ID and detailed docking scores are shown in Supplementary Data.

Coronaviruses are members of the Coronaviridae family. They are further subdivided into four genera: alpha-, beta-, gamma-, and delta- coronaviruses. The alpha- and beta- coronaviruses infect only mammals [1]. Noting that the 3D structure and the catalytic dyads of M^pro^ are conserved in alpha- and beta- coronaviruses, we employed molecular docking to predict the potent inhibition activities of Xanthohumol against various coronaviruses. A high-quality crystal structure of SARS-CoV-2 M^pro^ has been reported earlier [10], and the crystal structures of SARS-CoV, MERS-CoV, PEDV, and TGEV M^pro^ have been explored as well [30,31,32,33]. As shown in Figure 3A, Xanthohumol could be docked into the active pocket of a series of coronaviruses, M^pro^, and form hydrogen bonds and other molecular interactions. Most docking scores are between −6 to −8, which indicate that Xanthohumol presents excellent affinities with various M^pro^ structures (Figure 3B and Supplementary Data). To be noted, a hydrogen bond was found between Xanthohumol and the Cys-145 of SARS-CoV-2 M^pro^ that was essential to M^pro^ catalytical activities, explaining that Xanthohumol inhibited SARS-CoV-2 efficiently. It indicated that Xanthohumol is a potent drug candidate for further drug development.

### 2.4. Xanthohumol Restricts SARS-CoV-2 and PEDV Replication in the Cell-Based Assay

**Figure 4:**
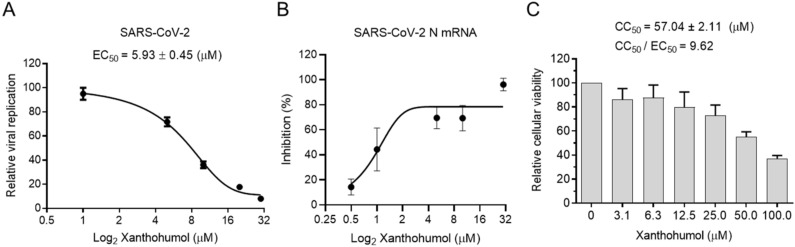
Xanthohumol inhibited SARS-CoV-2 replication in Vero-E6 cells. (**A**) Xanthohumol-inhibited SARS-CoV-2. Vero-E6 cells were pretreated with compounds at indicated concentrations for 1 h, and then infected with recombinant SARS-CoV-2 mNeonGreen virus (MOI = 0.5) for 24 h. The green fluorescence was scanned as described. The EC_50_ values of Xanthohumol were calculated and are shown. (**B**) Xanthohumol reduced viral RNA loads. SARS-CoV-2 N mRNA was measured with qRT-PCR and normalized to GAPDH. (**C**) Cytotoxicities of Xanthohumol. Data are shown as means ± SEM from three independent experiments.

Many chemicals presented significant inhibition efficiency in the enzymatic assay but failed in cell models because of their poor permeability, improper cellular metabolism, and other issues. We were wondering if Xanthohumol were able to hamper SARS-CoV-2 replication in cells. Calpeptin, a well-established cell-permeable cysteine protease inhibitor, was used as the positive control. Calpeptin significantly restricted SARS-CoV-2 infection (IC_50_ = 0.38 ± 0.01 μM), indicating that the infection model could properly reveal the inhibition activity of candidate compounds (Supplementary Figure S3A). To be noted, Xanthohumol dose-dependently inhibited SARS-CoV-2 in cells (Figure 4A,B), while it did not slow cell growth, and only negligibly reduced cellular viability at the high concentration (Figure 4C). In a plague assay, the IC_50_ value of Xanthohumol on SARS-CoV-2 was 5.93 ± 0.45 μM (Figure 4A). M^pro^ cleaves viral polyprotein to assemble viral replication machinery, which is critical for viral RNA duplication. This suggests that M^pro^ inhibitors could directly inhibit viral RNA replication by hampering the replication machinery assembly. To monitor whether Xanthohumol could inhibit SARS-CoV-2 RNA duplication by targeting its M^pro^, viral RNA loads of infected cells were also measured by qRT-PCR. This indicated that Xanthohumol could eliminate the viral genome effectively, which is highly consistent with the results from the plague assays mentioned above (Figure 4B). Moreover, the CC50 (the half cytotoxic concentration) value of Xanthohumol in Vero-E6 cells was 57.04 ± 2.11 μM, and the selection index was more than 9.5. This suggests that Xanthohumol is an outstanding lead compound for further developments.

**Figure 5:**
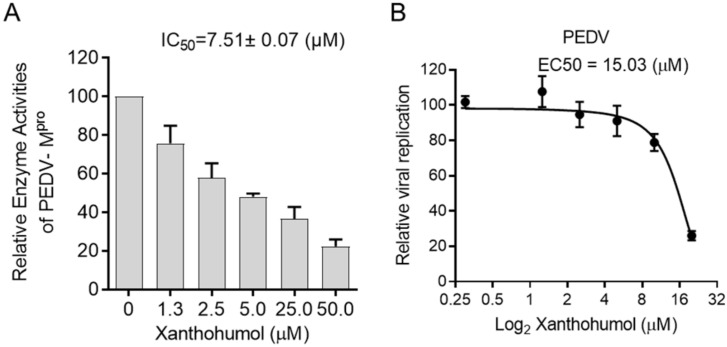
Xanthohumol inhibited PEDV M^pro^ and PEDV in vitro. (**A**) Xanthohumol inhibited PEDV M^pro^. (**B**) Xanthohumol inhibited PEDV replication in vitro. Vero-E6 cells were pretreated with the indicated concentration of Xanthohumol for 1 h and infected with PEDV (MOI = 1). Data are shown as means ± SEM from three independent experiments.

Considering the structural similarity between PEDV and SARS-CoV-2 M^pro^, we were wondering if Xanthohumol would inhibit PEDV M^pro^ enzymatic activity and then restrict PEDV replication. The enzymatic assay was performed as SARS-CoV-2, in which the PEDV M^pro^ was used instead of SARS-CoV-2 M^pro^. It indicated that Xanthohumol inhibited PEDV M^pro^ efficiently with an IC_50_ value of 7.51 ± 0.07 μM (Figure 5A). Moreover, Xanthohumol dose-dependently inhibited PEDV in Vero-E6 cells (Figure 5B).

## 3. Discussion

COVID-19 might recede soon. However, as the predicted Disease X by the WHO after the outbreak of SARS and MERS, coronaviral infections might routinely break out as the seasonal flu in the future. Moreover, coronaviruses are threatening livestock. It is still meaningful to screen safe, cheap, and broad-spectrum antiviral agents for coronaviruses. As shown in our study, Xanthohumol inhibited alpha- and beta- coronaviruses (Figure 4 and Figure 5), which contain all fatal pathogenic coronaviruses: PEDV, SARS-CoV, MERS-CoV, and the recent SRAS-CoV-2 [34]. As previously reported, Xanthohumol modulated inflammation and oxidative stresses [35], which might benefit infected individuals, rather than an antiviral agent. At this moment, Xanthohumol is an excellent lead compound for drug optimization, and more detailed studies are required for further developments.

The safety and tolerability in healthy adults of Xanthohumol has been well-established, and Xanthohumol is available as dietary supplements and ingredients in medical foods. Although it is impossible to get enough Xanthohumol from beers before being severely hurt by alcohol, because of its relatively high IC_50_ value against coronaviruses, it is hoped that Xanthumol might guard healthy individuals against the initiation of infection at a low concentration. Of course, more detailed systematic studies should be performed before the usage of Xanthohumol for any medicinal purpose. In this case, using Xanthohumol in nonalcohol foods and drinks may be an available strategy. Hops are cheap and easily available in some areas. Coronaviruses are heavily damaging our livestock industry and using hops as feed additives may also be suggested.

Xanthohumol had been found to be a broad-spectrum antiviral agent, presenting inhibition activities against many viruses, which include human cytomegalovirus (CMV), herpes simplex virus (HSV), human immunodeficiency virus (HIV), hepatitis C virus (HCV), and porcine reproductive and respiratory syndrome viruses (PRRSV) [36,37,38]. Recently, many researchers keep discussing the potent role of Xanthohumol or the crude extracts of hops in COVID-19 treatments [39,40]. However, it is still unknown whether Xanthohumol can inhibit coronaviruses, and the antiviral mechanism of Xanthohumol is elusive. It has been shown that its antioxidative activities could reduce viral-induced tissue damage, and Xanthohumol could also boost the antiviral activities of type I interferon [41,42]. In our study, we demonstrate that Xanthohumol target viral M^pro^ as a protease inhibitor. Interestingly, many protease inhibitors were clinically used for HIV treatments [43], of which Lopinavir, Indinavir, and Darunavir have been repurposed to inhibit SARS-CoV-2 M^pro^ [44]. The HCV NS3–4A protease drug, boceprevir, could inhibit SARS-CoV-2 replication by targeting M^pro^ in our and other groups’ independent studies [45,46]. To be noted, PRRSV belongs to Arteriviridae. Both Arteriviridae and Coronaviridea are members of Nidovirales. PRRSV has a similar assembly process of replicase as coronaviruses. It encodes a polyprotein that is be cleaved by viral proteins (also called main protease and Papain-like protease as SARS-CoV-2). This suggests that Xanthohumol might inhibit HIV, HCV, PRRSV, and coronaviruses via a similar mechanism, being a protease inhibitor.

As mentioned above, because of the large number of infected individuals, the high mutation rates, and the potent zoonotic feature of coronaviruses [4], the war against coronaviruses will be a long one. It is necessary to screen potent pan-inhibitors for the next unexpected outbreak. Herein, identifying a promised drug target becomes critical. Xanthohumol showed a pan-inhibitor activity against alpha- and beta- coronaviruses targeting M^pro^, and the GC-376 could inhibit SARS-CoV, SARS-CoV-2, and the Feline coronavirus (FcoV) via inhibiting M^pro^ [10,27,28]. A recently designed M^pro^ inhibitor also presented pan-inhibitor activities [10]. It will be interesting to screen M^pro^ inhibitors against coronaviral infection and to clarify whether M^pro^ is a promised drug target for the development of pan-inhibitors against various coronaviruses.

## 4. Materials and Methods

### 4.1. Cell Culture and Reagents

Vero-E6 cells (ATCC, VERO C1008 [Vero 76, clone E6, Vero E6] CRL-1586™) were cultured in Dulbecco’s modified Eagle’s medium (DMEM) supplement, with 10% fetal bovine serum (FBS), penicillin (100 IU/mL), and streptomycin (100 mg/mL). Cells were maintained in the incubator at 37 °C with 5% CO_2_. Candidate compounds were obtained from a chemical bank in the lab, which was originally purchased from TargetMol (Shanghai, China), or were synthesized by us. Xanthohumol, Calpeptin, Remdesivir, and MG132 were purchased from Selleck (Shanghai, China). We purchased 96-well black plates with transparent glass-bottoms from Cellvis (Shanghai, China).

### 4.2. Drug Treatment and Viral Infection

For cytotoxicity analysis, Vero-E6 cells (3000 cells/well) were seeded into 96-well plates. After 12 h, the cells were treated with the indicated chemicals at the final concentration, as shown. At 48-h post-treatment, 10 µL of resazurin (1 mg/mL) was added to each well and incubated for 3 h. The absorbance was measured on a SpectraMax i3 (Molecular Devices, San Jose, CA, USA).

For antiviral analysis, Vero-E6 cells were pretreated with Xanthohumol and Calpeptin for 1 h and infected with SARS-CoV-2 mNeonGreen virus (MOI = 0.5) [47]. At 24-h post-infection (hpi), the cells were fixed and scanned by Amersham Typhoon 5 (GE, Laurel, MD, USA). The fluorescence intensities were quantified by ImageJ (NIH). The mNeonGreen signals and the mRNA of the SARS-CoV-2 N gene were measured, as previously described [48]. For the PEDV analysis, cells were pretreated with Xanthohumol or Remdesivir for 1 h and then infected with PEDV (MOI = 1) in the presence of drugs for 24 h. Cells were harvested and fixed with 2% paraformaldehyde, permeabilized, and intracellularly stained with house-made mouse anti-PEDV nucleocapsid serum. Goat anti-mouse IgG, conjugated with Alexa Fluor 647 (Thermo Fisher, Waltham, MA, USA), was used as the secondary antibody. After two additional washes, cells were subjected to flow cytometry analysis (Thermo, Waltham, MA, USA) and data processing (FlowJo, Becton, Dickinson & Company, Franklin Lakes, NJ, USA). Porcine epidemic diarrhea virus (PEDV) was propagated in Vero-E6 cells [49,50].

### 4.3. Protein Expression, Purification, and Enzymatic Assay

Protein expression and enzymatical assays were performed as previously described, with minor modifications [51]. SARS-CoV-2 and PEDV M^pro^ were generated as described previously [51]. Protein purity was identified by Coomassie Brilliant Blue staining. The enzymatic activity of M^pro^ was measured by continuous kinetic assays using an identical fluorogenic substrate, MCA-AVLQ/SGFR-Lys (DNP)-Lys-NH2 (Apetide Co., Ltd., Shanghai, China), as previously described [51] (Supplementary Figure S2B). The fluorescence intensities were monitored by a microplate reader (SpectraMax i3x, Molecular Devices). The excitation and emission wavelengths were 320 and 405 nm, respectively. Experiments were performed in 100 μL buffer (50 mM Tris-HCl, 1 mM EDTA, pH 7.3) containing 100 nM M^pro^, 2 µM substrate, and 1 µL of the desired concentration of drugs. The compounds and the M^pro^ were incubated at RT for 15 min. Reaction velocities were analyzed by SoftMax Pro. EC_50_, Km, and Ki were calculated by GraphPad Prism6.

### 4.4. Molecular Docking

The M^pro^ structure information was obtained from the PDB databank (SARS-CoV-2: 6lu7; SARS-CoV: 3sn8; MERS-CoV: 4rsp; PEDV: 5gwz; TGEV: 1p9u). In addition, 378 of the M^pro^ structure information, analyzed by different research groups, was also obtained from the PDB database. The details are available in SI1. The structures of the proteins were prepared via the Protein Preparation Wizard task from Schröndinger for structure optimization. The structures of the compounds were prepared by LigPrep of Schrödinger (Schrödinger), and docked into the M^pro^ substrate-binding domain by AutoDock Vina. The docking score was the top score of the different docked modes. The M^pro^ structural superposition was analyzed by PyMol alignment.

## Data Availability

The data presented in this study are available upon request from the corresponding authors.

## Supplementary Materials

The Supplementary Materials are available online at https://www.mdpi.com/article/10.3390/ijms222212134/s1.
