# Supplementary material for: Xanthohumol Is a Potent Pan-Inhibitor of Coronaviruses Targeting Main Protease
Source: Int J Mol Sci. 2021 Nov 9;22(22):12134. doi: 10.3390/ijms222212134 (PMC8624673; doi:10.3390/ijms222212134)
Supplement: Supplementary file 1 [file ijms-22-12134-s001.zip › ijms-1419558-sm/Supporting Information.pdf]

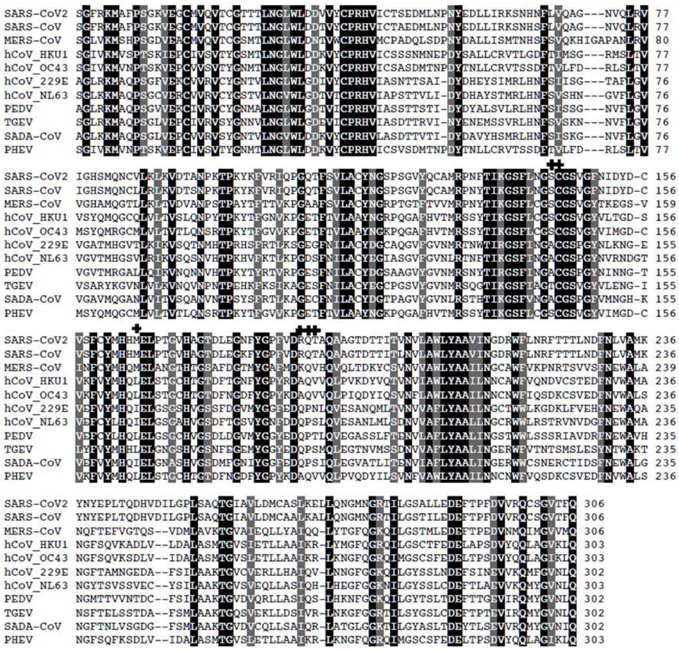

Fig. S1 The amino acid sequences are consistent in pathogenic coronaviruses.

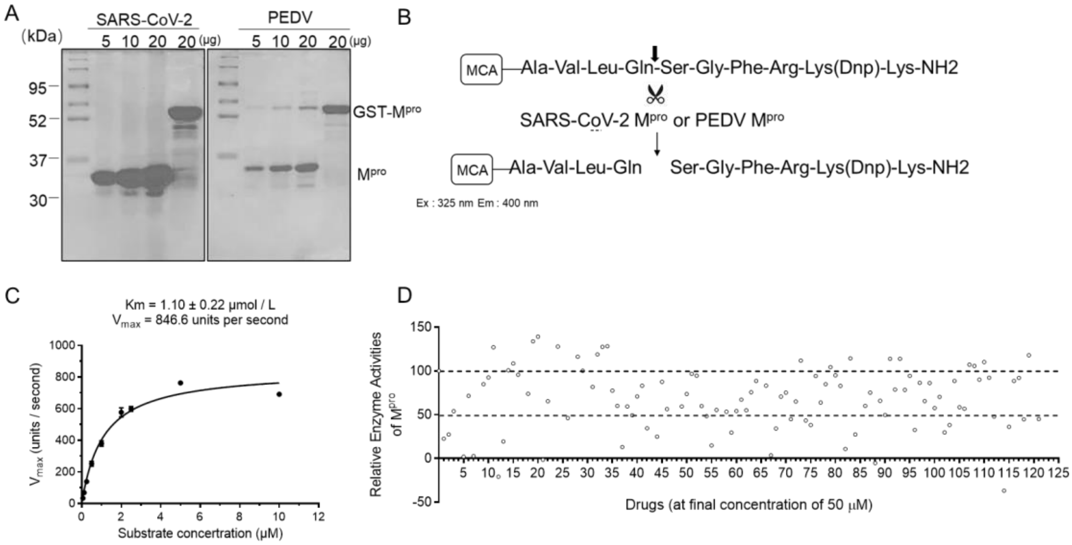

Fig. S2 In vitro enzyme activity assay. 100 nM purified M^{pro} (A) and different concentration of fluorescent labeled substrate (B) were mixed and the fluorescent signal were monitored. Km and V_{max} were calculated and showed in (C). Data were shown as means ± SEM from three independent experiments. (D) Relative enzyme activities of M^{pro} after 50 μM compounds treatment. The results of some compounds with severe signal quenching have been removed.

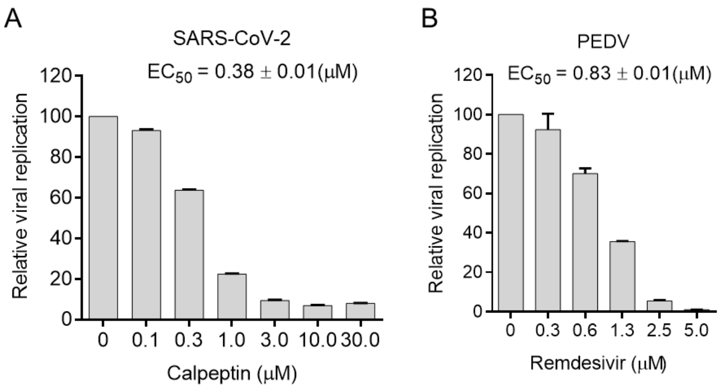

Fig. S3 Calpeptin and Remdesivir inhibited SARS-CoV-2 and PEDV replication in Vero-E6 cells, respectively.
